# Diagnosis of Aspergillus Osteomyelitis of the Clivus and Sella Turcica in a Patient With Type 2 Diabetes and a History of Prolonged Intranasal Corticosteroid Use

**DOI:** 10.7759/cureus.78779

**Published:** 2025-02-09

**Authors:** Rushi Patel, Veronika Kholodovych, Miguel Tellado-Fente, Amy Vittor

**Affiliations:** 1 Internal Medicine, Malcom Randall Veterans Affairs Medical Center, University of Florida College of Medicine, Gainesville, USA; 2 Infectious Diseases and Global Medicine, Malcom Randall Veterans Affairs Medical Center, University of Florida College of Medicine, Gainesville, USA; 3 Pathology, Malcom Randall Veterans Affairs Medical Center, University of Florida College of Medicine, Gainesville, USA

**Keywords:** aspergillus fumigatus, aspergillus osteomyelitis, atypical skull-base osteomyelitis, diabetes, diabetes type 2, fungal osteomyelitis, intranasal corticosteroids, sinusitis, voriconazole

## Abstract

A 69-year-old immunocompetent male with uncontrolled type 2 diabetes mellitus (T2DM) presented with atypical left-sided headaches, diverging from his usual migraine pattern. Historically experiencing right-sided migraines, the patient's shift to left-sided headaches occurred after a month of using fluticasone for cold-like symptoms and potential mold exposure at home. Computed tomography (CT) and magnetic resonance imaging (MRI) suggested the diagnosis of skull base osteomyelitis. Endoscopic sphenoidotomy revealed Aspergillus species, leading to treatment with voriconazole. Despite the rarity of skull base Aspergillus osteomyelitis in patients who are not classically immunocompromised, this case underscores its possibility, especially in the context of diabetes and prolonged corticosteroid use. Similar literature is limited but highlights the high fatality rate of invasive fungal infections in diabetic patients and the complexity of diagnosing skull base osteomyelitis due to its varied presentations. Management involved surgical debridement and systemic antifungal therapy. This case aims to add to the limited literature on cranial Aspergillus osteomyelitis, advocating for heightened clinical vigilance, a multifaceted approach involving prompt evaluation, surgical intervention, and tailored antifungal therapy. The case highlights the need for considering fungal etiologies in atypical headache presentations and emphasizes multidisciplinary management for favorable outcomes in an otherwise morbid condition.

## Introduction

Aspergillus osteomyelitis, a form of invasive fungal infection, often affects severely immunocompromised and immunodeficient individuals, making its occurrence an unusual and noteworthy event [1-4]. Furthermore, the etiology of Aspergillus osteomyelitis in the craniofacial skeleton, particularly involving the clivus and sella turcica, remains an area of clinical rarity. 

Diagnosis of invasive fungal infections in immunocompromised individuals poses a challenge due to the difficulty of distinguishing between invasive and colonizing features in the setting of poor sensitivity and specificity with current diagnostic methods and a prolonged amount of time for diagnostic studies to return [5-7]. 

This case report presents an instance of the diagnosis of Aspergillus osteomyelitis involving the clivus and sella turcica in a 69-year-old male with a background of uncontrolled type 2 diabetes, well-controlled other chronic conditions, and no evidence of additional immunosuppression or immunodeficiency. This report discusses the intricate interplay of factors contributing to this rare diagnosis, including prolonged use of fluticasone, uncontrolled diabetes, and potential environmental exposures. Through this case, we aim to contribute to the growing body of literature on rare skull base Aspergillus osteomyelitis in individuals who are not classically immunocompromised, providing insights into its diagnosis, management, and clinical course. The rarity of this condition in such patients underlines the significance of our report in expanding the current understanding of Aspergillus infections beyond conventional paradigms.

## Case presentation

A 69-year-old male with a past medical history of chronic migraines, hypertension, hyperlipidemia, Type 2 Diabetes Mellitus (T2DM), osteoarthritis, gastroesophageal reflux disease, and erectile dysfunction, presented to the emergency department (ED) with atypical left-sided headaches, deviating from his usual migraine pattern. 

Historically, the patient's migraines were unilateral, only affecting the right side, characterized by eye pressure, sinus congestion, and subsequent diffuse unilateral head pressure, reliably alleviated by sumatriptan. He reported his symptoms have been consistent for the past thirty-five years. Approximately one month before his ED presentation, he was treated at an urgent care facility for cold-like symptoms without antibiotics and prescribed fluticasone nasal spray for congestion. Despite continuous use of fluticasone for one month, his symptoms persisted. He reported potential mold exposure at home with recent bathroom renovations, as well as a history of uncontrolled T2DM, with an HgA1c of 8.2%, although his other chronic conditions were well-controlled. 

Despite sumatriptan administration, his headache persisted. He denied accompanying fever, chills, nausea, vomiting, otic, or neurologic symptoms. In the ED, the patient was hypertensive with a blood pressure of 186/81 mmHg; he was otherwise afebrile, and the rest of his vitals were within normal limits. His initial labs are reported in Table 1.

**Table 1:** Admission and follow-up laboratory values

Notable labs	Patient value	Reference range
White blood cell count	4.71 k/cmm	4.6-10.8 k/cmm
Hemoglobin	11.6 g/dL	13.9-18 g/dL
Platelet count	259 k/cmm	130-440 k/cmm
Erythrocyte sedimentation rate	100 mm/hr	0-15 mm/hr
C-reactive protein	1.1 mg/dL	<0.5-0.5 mg/dL
Hemoglobin A1c	8.2%	
Follow-up labs		
Erythrocyte sedimentation rate	60 mm/hr	0-15 mm/hr
C-reactive protein	0.9 mg/dL	<0.5-0.5 mg/dL

**Figure 1:**
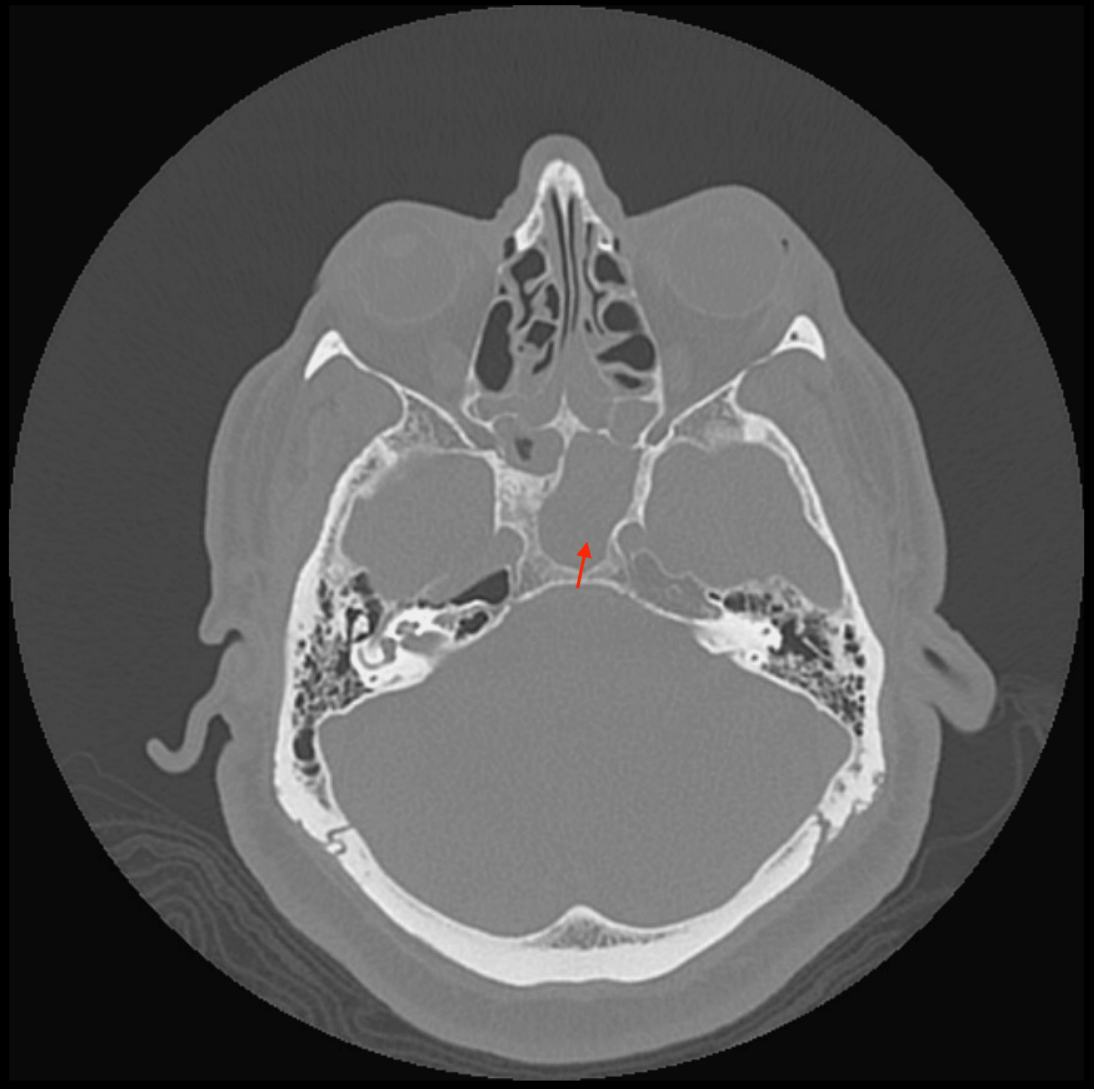
CT widening of the left sphenoid ostium, with thinning and sclerosis of the surrounding sphenoid sinus/clivus (arrow) CT: Computed tomography

His physical exam was unremarkable, specifically, he was without vision abnormalities, orbital edema, ophthalmoplegia, cranial nerve, or focal neurologic deficits. His ear exam was unremarkable, bilateral ears were without lesions or inflammation, and with intact translucent tympanic membranes. Computed tomography (CT) of the head without contrast revealed a widening of the left sphenoid ostium, with thinning and sclerosis of the surrounding sphenoid sinus/clivus as noted in Figure 1.

**Figure 2:**
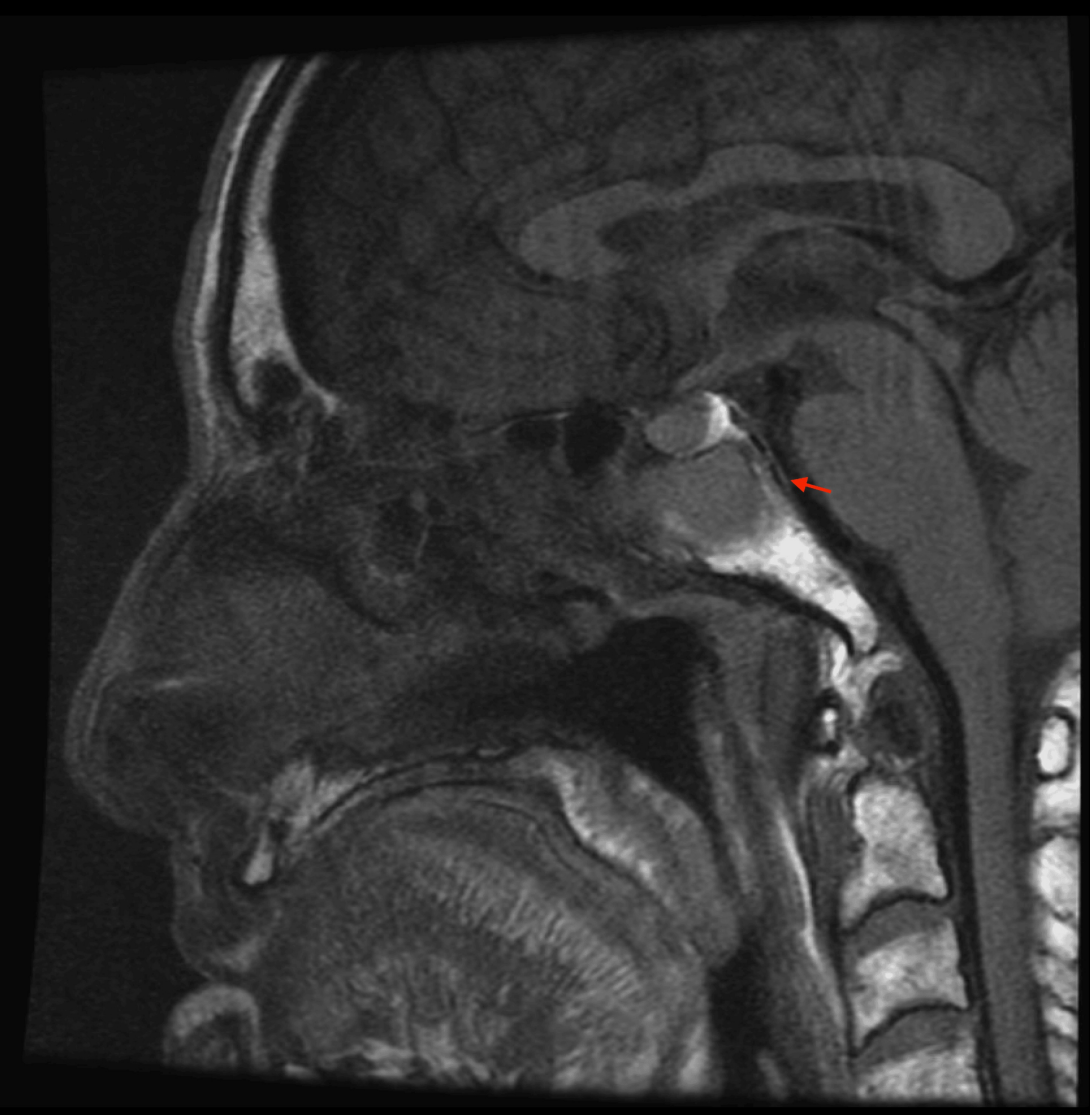
MRI with and without contrast of the orbit demonstrated abnormal T1 marrow suppression within the anterior superior margin of the clivus/sella turcica (arrow), concerning osteomyelitis, along with purulent debris filling the left sphenoid sinus and extruding through the sphenoid ostium (per radiologist's read) MRI: magnetic resonance imaging

Magnetic resonance imaging (MRI) with and without contrast of the orbit demonstrated abnormal T1 marrow suppression within the anterior superior margin of the clivus/sella turcica, concerning osteomyelitis, along with purulent debris filling the left sphenoid sinus and extruding through the sphenoid ostium as noted in Figure 2.

**Figure 3:**
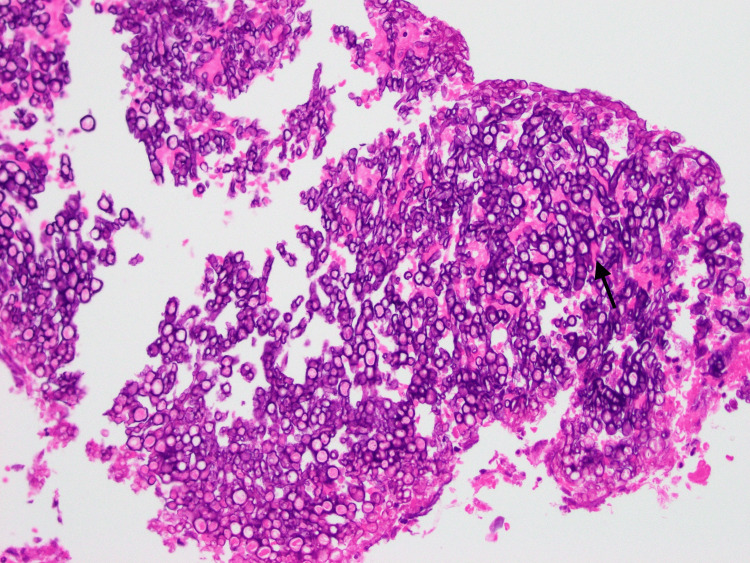
Medium-power hematoxylin and eosin-stained tissue slide of left sphenoid and left sinus debris, notable for numerous organisms demonstrating hyphae (arrow), consistent with Aspergillus species Histopathology image courtesy: Dr. Miguel Tellado-Fente, Malcom Randall Veterans Affairs Department of Pathology.

**Figure 4:**
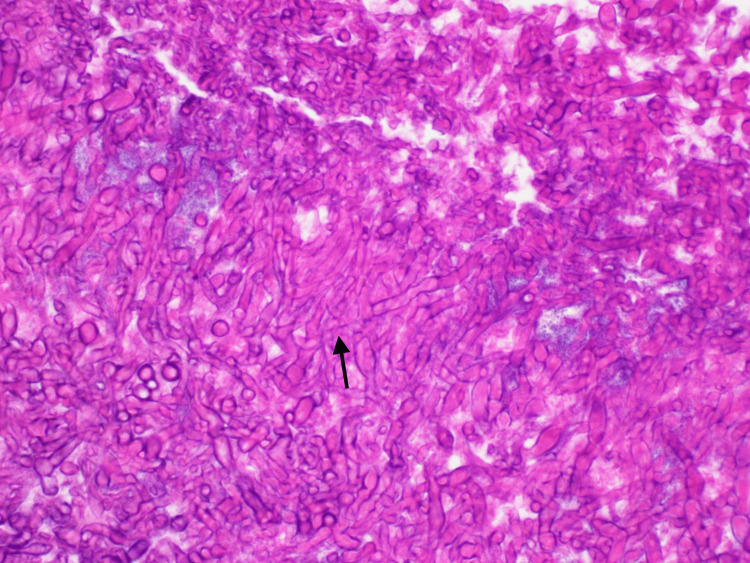
High-power hematoxylin and eosin-stained tissue slide of left sphenoid and left sinus debris, notable for numerous organisms demonstrating acute-angle branching hyphae (arrow), consistent with Aspergillus species Histopathology image courtesy: Dr. Miguel Tellado-Fente, Malcom Randall Veterans Affairs Department of Pathology.

Ear, nose, and throat (ENT) specialists were consulted and the patient was started empirically on vancomycin and piperacillin/tazobactam. He underwent endoscopic sphenoidotomy, revealing inflammation, mucosal edema of the left sphenoid, and a single polyp, which was removed and sent for histopathology. In addition, “fungal debris” was grossly noted in the left sphenoid sinus, per ENT assessment. Aerobic and anaerobic bacterial cultures were submitted and later resulted in normal nasal flora. No fungal cultures were submitted. Surgical pathology of the nasal polyp revealed fungal hyphae demonstrating acute-angle branching consistent with Aspergillus species (Figures 3, 4), leading to the cessation of intravenous antibiotics and initiation of oral voriconazole 200mg twice daily with therapeutic dose monitoring to target a therapeutic trough concentration of 1-5.5 mg/mL, with plan to treat for at least three months with interval repeat imaging. Of note, the Aspergillus galactomannan serum antigen test and β-d-Glucan serum assay were not sent as we felt the diagnosis was sufficiently made based on clinical factors, radiology, and pathology. 

The rest of his hospital course was unremarkable, and he was discharged with follow-up plans with ENT, infectious diseases, and primary care. 

At his one-week hospital discharge appointment with ENT, the patient reported a resolution of headache and sinus symptoms. During that visit, a fiberoptic endoscopic exam was performed with no debris seen in the left sphenoid sinus, at the site of original debris. He was then discharged from routine ENT clinic visits. At six weeks post-hospital discharge, the patient had a clinic visit with infectious diseases during which he continued to be symptom-free. Three months later, a repeat MRI revealed no marrow abnormalities, specifically, no concerning change in the pattern of signal intensity within the clival marrow, and no imaging features suggestive of skull base infection or aggressive sinusitis. Follow-up labs are demonstrated in Table 1. Of note, voriconazole levels were measured every two weeks throughout the duration of the patient’s course and remained consistently in the therapeutic range. Given the radiographic resolution of acute skull base osteomyelitis and clinical improvement, voriconazole was stopped. The patient was clinically doing well without antifungal therapy at his six-month post-discharge follow-up with no evidence of relapse. 

## Discussion

Aspergillus osteomyelitis of the skull base is a cause of atypical skull base osteomyelitis and a rare and potentially life-threatening condition [1,2]. Our case of a 69-year-old with uncontrolled T2DM presenting with *Aspergillus* osteomyelitis of the clivus and sella turcica highlights significant challenges in the diagnosis and management of atypical skull base osteomyelitis in this patient population [3-5]

Atypical skull base osteomyelitis remains a challenging diagnosis due to its rarity and varied clinical presentation [5]. Although our patient did not have any focal neurologic deficits, other patients have presented with multiple cranial nerve deficits, such as proptosis and diplopia mediated by deficits to cranial nerve three, or slurred speech and dysphagia mediated by deficits to cranial nerve twelve, mimicking other severe neurological conditions or malignancies [5,6]. The absence of clear epidemiological data on the frequency and symptomatology of atypical skull base osteomyelitis further adds to the complexity of clinical decision-making [1-3]. 

The most common cause of atypical skull base osteomyelitis is a paranasal sinus infection, although extension of infection from the face, oral cavity, and pharynx have been described [5,6]. In this case, the findings described as "fungal debris" on the fiberoptic nasal endoscopy coupled with radiographic findings, and histopathology confirmed the suspicion of fungal osteomyelitis, with Aspergillus species being the most likely according to the histopathologist. The most common species is *Aspergillus fumigatus*, followed by *Aspergillus glavus,* and *Aspergillus nidulans* [2]. In our case, aerobic and anaerobic bacterial cultures were noncontributory and no additional culture data was available, emphasizing the importance of submission of designated fungal cultures for identification of the fungal organism and its antifungal susceptibility. In addition, broad-range PCR was not able to be sent due to a lack of additional specimens but would be of value in a similar case for organism identification. Notably, the Aspergillus galactomannan serum antigen test and β-d-Glucan serum assay were not sent to our patient, and could also be helpful in diagnosis, and if elevated, management, as these serum tests could be easily repeated and trended in the outpatient setting. 

Misdiagnosis or delayed diagnosis of atypical and fungal skull base osteomyelitis can have fatal outcomes [6-8]. In patients with T2DM, typical skull base osteomyelitis is commonly associated with otic infections and mediated by bacteria such as *Pseudomonas aeruginosa* and *Staphylococcus aureus *[5]. A review of Aspergillus osteomyelitis cases reported a notable proportion of patients having underlying hematological malignancies, prolonged neutropenia, history or organ transplantation as well as diagnosed immunodeficiencies such as chronic granulomatous disease and Th17 deficiency [2,4]. We suggest that the presence of uncontrolled diabetes in addition to intra-nasal corticosteroid use should pose a high index of suspicion for atypical skull base osteomyelitis with fungal etiologies, especially in patients presenting with prolonged atypical cranial symptoms [3,5].

Management of atypical skull base osteomyelitis and Aspergillus osteomyelitis typically involves a combination of surgical debridement and antimicrobial therapy [1,2,9]. Our approach, comprising surgical debridement alongside systemic antifungal agents such as voriconazole, aligns with established strategies, emphasizing the importance of early and aggressive treatment [6,7,9]. Ultimately, the selection of oral voriconazole for this case was based on the patient's clinical stability and lack of neurologic deficit. However, clinically unstable patients or those with particularly extensive fungal involvement, neurologic compromise, and/or joint involvement may benefit from initiation of intravenous amphotericin with or without combination with voriconazole for initial stabilization and control, especially while identification of the infecting pathogen and its antifungal susceptibility is in progress [1,8,9]. 

## Conclusions

Aspergillus osteomyelitis in an immunocompetent patient is rare. However, this case of a diabetic patient who used fluticasone for one month prior highlights the importance of considering atypical risk factors for fungal infections in skull base osteomyelitis. It also emphasizes the need for an individualized and multidisciplinary approach to treatment, tailored to the patient’s clinical stability and underlying conditions. This case underscores the critical role of multidisciplinary management, including early surgical intervention, collection of diagnostic and microbiologic data, and targeted antifungal therapy, along with appropriate follow-up, in achieving favorable outcomes in an otherwise morbid disease.
